# Clinicopathological features, survival and risk in breast cancer survivors with thyroid cancer: an analysis of the SEER database

**DOI:** 10.1186/s12889-019-7947-y

**Published:** 2019-11-29

**Authors:** Shuting Li, Jiao Yang, Yanwei Shen, Xiaoai Zhao, Lingxiao Zhang, Biyuan Wang, Pan Li, Yunmei Wang, Min Yi, Jin Yang

**Affiliations:** 1 Department of Medical Oncology, the First Affiliated Hospital of Xi’an Jiaotong University, 277 West Yanta Road, Xi’an, 710061 Shaanxi China; 2 Department of Medical Oncology, Shaanxi Provincial Tumor Hospital, Xi’an, China; 3 Department of Translational Medicine, the First Affiliated Hospital of Xi’an Jiaotong University, Xi’an, China

**Keywords:** Breast cancer, Thyroid cancer, Clinicopathological features, Survival, Standardized incidence ratios

## Abstract

**Background:**

The co-occurrence of breast cancer (BC) and thyroid cancer (TC) has been mentioned for several years, researchers observed an increased risk of BC patients to develop TC, but few researches concern about the features, survival of BC patients followed by TC and the influent factors of the incidence risk. The present study aimed to estimate the clinicopathological features, survival of BC survivors who had primary TC and the predictive factors on the risk of BC patients to develop TC.

**Methods:**

Women diagnosed with BC between 1992 and 2011, and then developed TC from the Surveillance, Epidemiology, and End Results Database were included. Standardized incidence ratios (SIRs) was used to perform multiple primary analyses, generated from the multiple primary-SIR program in SEER*Stat.

**Results:**

A total of 842 BC then TC patients were included, the median age was 54 years. Additionally, 78.39% were white, 60.45% had T1 cancer, 62.47% had negative lymph nodes, and more than 75% had infiltrating duct carcinoma, 5-year survival rate was 95.4%. Compared with BC only patients, they were younger, had smaller tumor size and a relatively better prognosis. The risk of developing TC was higher in BC patients than in the general population (SIR 1.22, 95% CI [1.14, 1.31]), especially within 3 years. The influent factors of SIR were black race, BC tumor site, grade and ER/PR positive expression.

**Conclusions:**

BC patients followed by TC had its particular clinicopathological features. Compared with the features and survival of BC only patients, they were younger, had a smaller tumor size and a relatively better prognosis. Furthermore, BC patients had a high risk of developing TC, especially within 3 years. Black women, primary tumor located in an upper-outer, central, or overlapping site, high grade tumor and with positive hormone receptor expression were predictive factors to develop TC.

## Introduction

According to the 2012 GLOBOCAN statistics, breast cancer (BC) is the second most common cancer overall and the most frequently diagnosed cancer in women, with 1.67 million women diagnosed with BC worldwide [1]. Because of the high rate of survival and the relatively young age at diagnosis, the number of BC survivors is increasing, and therefore, there is a chance of experiencing other cancers. Five most common cancers occurred in BC survivors are contralateral breast cancer (cumulative incidence at 8 years is 1.64%), urinary system cancer (0.43%), lung cancer (0.31%), thyroid cancer (0.28%) and melanoma (0.21%) [2]. Several articles have suggested that BC and thyroid cancer (TC) can occur together in female patients, and the rate of occurrence is much higher than that expected by chance [3]. Studies have evaluated the risk of developing second primary malignancies in patients with BC or TC history [2, 4–6], an increased risk of developing TC in BC survivors was observed. It is suspected that BC and TC share some common etiological factors [7–9], and some treatment-related factors may play a role in its co-occurrence. Besides, study on the comparison of features and prognosis between BC patients with TC and TC only patients was conducted [10]. However, few studies focused on the differences on clinicopathological features and prognosis between BC survivors with TC and BC only patients, the factors that influence the risk of BC survivors to develop TC is unclear.

The present study aimed to estimate the clinicopathological features, survival, risk and influent factors of SIRs on BC survivors followed by primary TC by analyzing the Surveillance, Epidemiology, and End Results (SEER) Database.

## Materials and methods

### Data source

Data were obtained from 13 US registration centers in the SEER Database, a database programed by US National Cancer Institute, which collects, processes, and provides data on approximately 10% of the US population, using the SEER*Stat software program (version 8.3.2; http://seer.cancer.gov/seerstat; accessed April 14, 2016) under a user agreement. In order to obtain data of BC patients who subsequently developed TC and had the follow-up records for at least 2 years until 2013, we used the database to identify patients whose primary tumor site codes were C50.0–C50.9, and histology codes were 8500/3 (infiltrating duct carcinoma), 8520/3 (lobular carcinoma), 8521/3 (infiltrating ductular carcinoma), 8522/3 (infiltrating duct and lobular carcinoma), 8523/3 (infiltrating duct mix with other types of carcinoma), 8524/3 (infiltrating lobular mix with other types of carcinoma), and 8541/3 (Paget disease and infiltrating ductal carcinoma of breast) from 1992 to 2011. Considering the opinion accepted by most scholars that occurrence of 2 types of cancers within 6 months is mainly considered as simultaneous occurrence, the latency between BC and TC was required at least 6 months. BC only cohort also met the site codes, histology codes and follow-up years criteria mentioned above, but the number of primary malignancies was only one.

A total of 842 cases matched the criteria and composed of BC then developing TC cohort, 332,424 patients composed of BC only cohort. All patients were followed until last known follow-up, death, or December 31, 2013, whichever occurred first. Data, including basic patient information, clinical features, pathologic characteristics, standard incidence rates, and survival information, were collected.

### Statistical methods

Overall survival (OS) was defined as the time from the diagnosis of BC to the time of death from any cause or loss of contact. OS curves were calculated using the Kaplan-Meier method. Cox proportional hazards models were used to evaluate the influence of collected clinicopathological factors on OS of BC then TC patients. Different factors have different impact on patients’ survival, after adjusting for the influence of other factors, a certain factor may have individual impact on survival, reflecting on the *p* value<0.05 in Cox proportional hazards models, this factor was known as a prognostic factor. The standardized incidence ratios (SIRs) was used to perform multiple primary analyses, generated from the multiple primary-SIR program in SEER*Stat (version 8.3.2; April 14, 2016). SIR was used to compare the incidence rate for the cohort of patients previously diagnosed with BC and subsequently diagnosed with TC to that expected in the general population. SIR = standardized incidence rate of BC patients to develop TC / standardized incidence rate of general population to develop TC. SIR> 1 means BC patients have more risk to develop TC than general women, factors with a SIR> 1 and *p* value<0.05 was known as a predictive factor. Statistical analyses were performed using SPSS version 21.0 (IBM Corp., Armonk, NY, USA). All statistical tests were two-tailed, and *p* < 0.05 was considered significant.

## Results

### Differences between the clinicopathological features of BC then TC group and BC only group

Clinicopathological features of BC then TC group and BC only group are presented in Table 1. With regard to the features of BC patients followed by TC, more than half of the patients were more than 50 years when diagnosed with BC, and the largest age group was 51–60 years, only 11.28% of the BC survivors with primary TC were diagnosed before 40 years of age. Regarding race, white women represented 78.39% of the patients, which mainly due to the composition of the population. Regarding other pathological features, more than 1/3 of the patients had BC in the upper-outer quarter, 60.45% had T1 cancer, 62.47% had negative lymph nodes, more than 3/4 had infiltrating duct carcinoma, more than 1/3 had grade 2 or 3 histology, and more than half were hormone receptor-positive cancer. Compared these features with those of BC only patients, some particular features were obtained. BC patients followed by TC showed a significantly younger median age at diagnosis (54 vs 59, *p* < 0.001) and accordingly had more patients of < 50 years group (37.41% vs 28.58%). Considering tumor features, BC sizes between two groups were significantly different, BC patients followed by TC had more small size (T ≤ 20 mm) tumors.

**Table 1:** Clinicopathological features of 842 breast cancer patients followed by thyroid cancer and 332,424 breast cancer only patients

	Breast cancer then develop thyroid cacer, *n* = 842		Breast cancer only, *n* = 332,424		*p*-value
	*n*	%	*n*	%	
Age at diagnosis					< 0.001
Median age (years)	54 [47, 63]		59 [49, 71]		
≤ 40	95	11.28%	25,787	7.76%	
41–50	220	26.13%	69,214	20.82%	
51–60	257	30.52%	80,754	24.29%	
61–70	172	20.43%	70,475	21.20%	
> 70	98	11.64%	86,194	25.93%	
Race					0.106
White	660	78.39%	267,366	80.43%	
Black	77	9.14%	30,469	9.17%	
American Indian	6	0.71%	2267	0.68%	
Asian or Pacific Islander	97	11.52%	30,311	9.12%	
Unknown	2	0.24%	2011	0.60%	
Tumor site					0.912
Central portion	55	6.53%	18,578	5.59%	
Nipple	3	0.37%	1889	0.66%	
Upper-inner	90	10.69%	34,580	10.40%	
Lower-inner	44	5.22%	17,684	5.32%	
Upper-outer	301	35.75%	116,708	35.11%	
Lower-outer	56	6.65%	22,500	6.77%	
Overlapping lesion	167	19.83%	68,460	20.59%	
Unknown	126	14.96%	52,033	15.65%	
Tumor size					< 0.001
T ≤ 20 mm	509	60.45%	186,656	56.15%	
21 mm < T ≤ 50 mm	235	27.91%	88,752	26.70%	
T > 50 mm	39	4.63%	14,467	4.35%	
Unknown	59	7.01%	42,553	12.80%	
Positive lymph nodes					0.358
0	526	62.47%	198,449	59.70%	
1–3	174	20.66%	71,560	21.53%	
4–9	55	6.53%	22,700	6.83%	
≥ 10	27	3.21%	14,593	4.38%	
Unknown	60	7.13%	25,127	7.56%	
Histology					0.650
Infiltrating duct carcinoma	689	81.83%	267,864	80.57%	
Lobular carcinoma	71	8.43%	29,570	8.90%	
Mixed	82	9.74%	34,993	10.53%	
Grade					0.351
1	136	16.15%	56,672	17.05%	
2	350	41.57%	130,229	39.18%	
3	283	33.61%	111,645	33.58%	
4	13	1.54%	4636	1.39%	
Unknown	60	7.13%	29,247	8.80%	
ER					0.908
Positive	601	71.38%	234,140	70.43%	
Negative	155	18.41%	62,340	18.76%	
Borderline	2	0.24%	1062	0.32%	
Unknown	84	6.41%	34,882	10.49%	
PR					0.090
Positive	532	63.18%	197,737	59.48%	
Negative	202	24.00%	92,509	27.83%	
Borderline	6	0.71%	2069	0.62%	
Unknown	102	12.11%	40,113	12.07%	
HER-2					0.061
Positive	13	1.55%	5966	1.80%	
Negative	45	5.34%	32,120	9.66%	
Unknown	784	93.11%	294,341	88.54%	

*ER* Estrogen receptor, *PR* Progesterone receptor, *HER-2* Human epidermal growth factor receptor 2

### Survival and survival influent factors of BC patients followed by TC

Kaplan-Meier curves were used to demonstrate the OS curves. The 5-year OS rate of BC then TC patients was 95.4%, BC only patients was 88.9%.

Cox proportional hazards models were used to evaluate the influence of collected clinicopathological factors on OS of patients developed TC after BC diagnosis, results are summarized in Table 2. After adjusting for the influence of other factors, *p* value<0.05 in Cox proportional hazards models indicates this factor as a prognostic factor, which means BC then TC patients with this feature have an increased probability to get a worse prognosis. In Table 2, After adjusting for the influence of other factors, the age at diagnosis of BC (*p* < 0.001) and the number of positive lymph nodes (*p* < 0.001) were prognostic factors of BC patients followed by TC. With regard to age, using ≤40 years as a reference, 61–70 and > 70 age groups had a relatively worse prognosis. With regard to lymph nodes, when zero positive metastatic lymph nodes was used as a reference, with the increase in the number of positive metastatic lymph nodes, the shorter OS was obtained.

**Table 2:** Cox proportional hazards analysis on overall survival of 842 breast cancer patients followed by thyroid cancer

Variable	HR (CI)	*p*-value
Age at diagnosis		< 0.001
≤ 40	Reference	
41–50	1.158 (0.519–2.582)	0.720
51–60	1.613 (0.737–3.533)	0.232
61–70	4.940 (2.306–10.585)	< 0.001
> 70	11.681 (5.409–25.225)	< 0.001
Race		0.279
Tumor site		0.520
Tumor size		0.096
Positive lymph nodes		< 0.001
0	Reference	
1–3	1.649 (1.049–2.591)	0.030
4–9	2.440 (1.235–4.821)	0.010
≥ 10	6.515 (3.106–13.663)	< 0.001
Unknown	2.054 (1.200–3.417)	0.009
Histology		0.125
Grade		0.321
ER		0.831
PR		0.767

*HR* Hazard ratio, *CI* Confidence interval, *ER* Estrogen receptor, *PR* Progesterone receptor

**Fig. 1:**
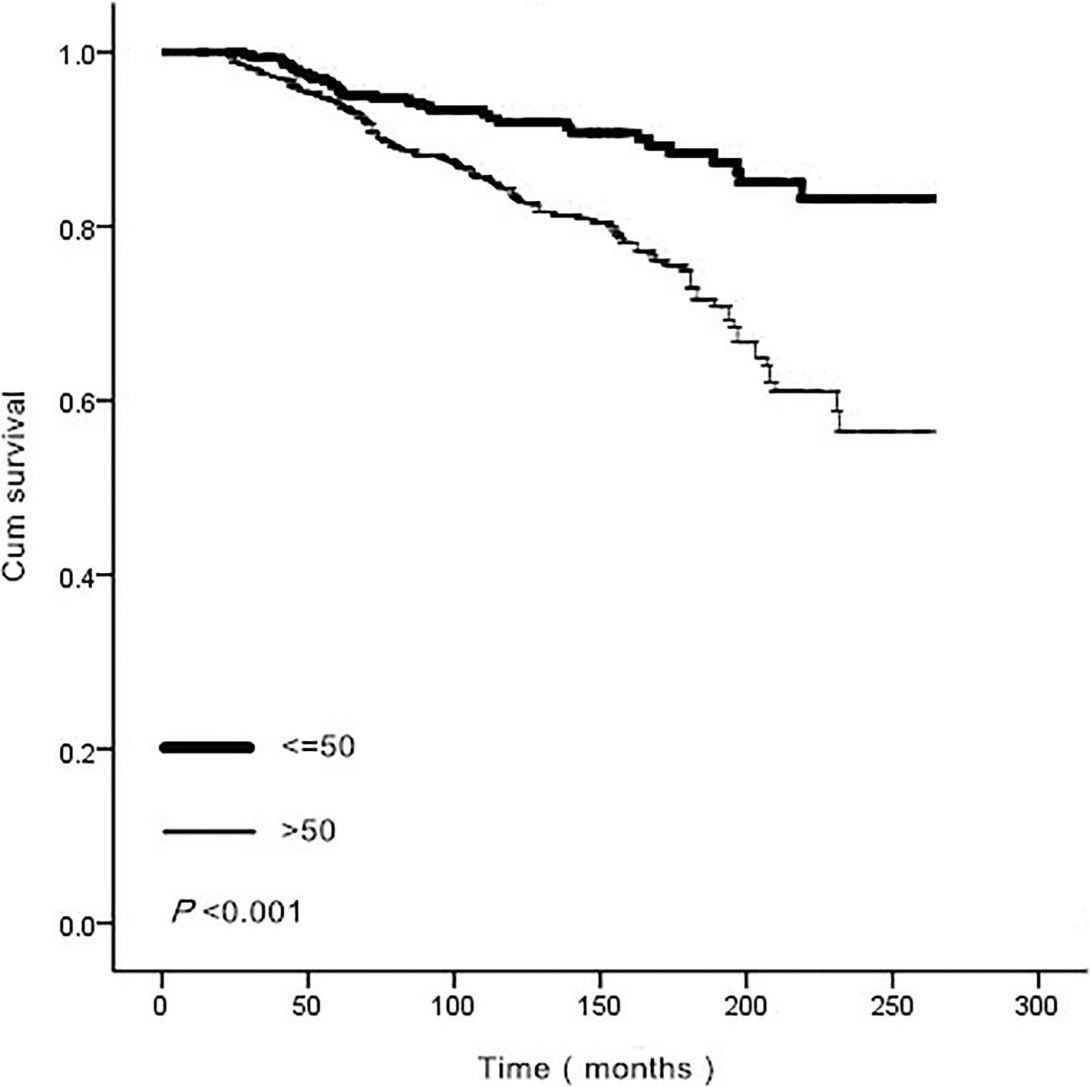
Survival curves for an age 50 cut-off of 842 breast cancer patients followed by thyroid cancer

**Fig. 2:**
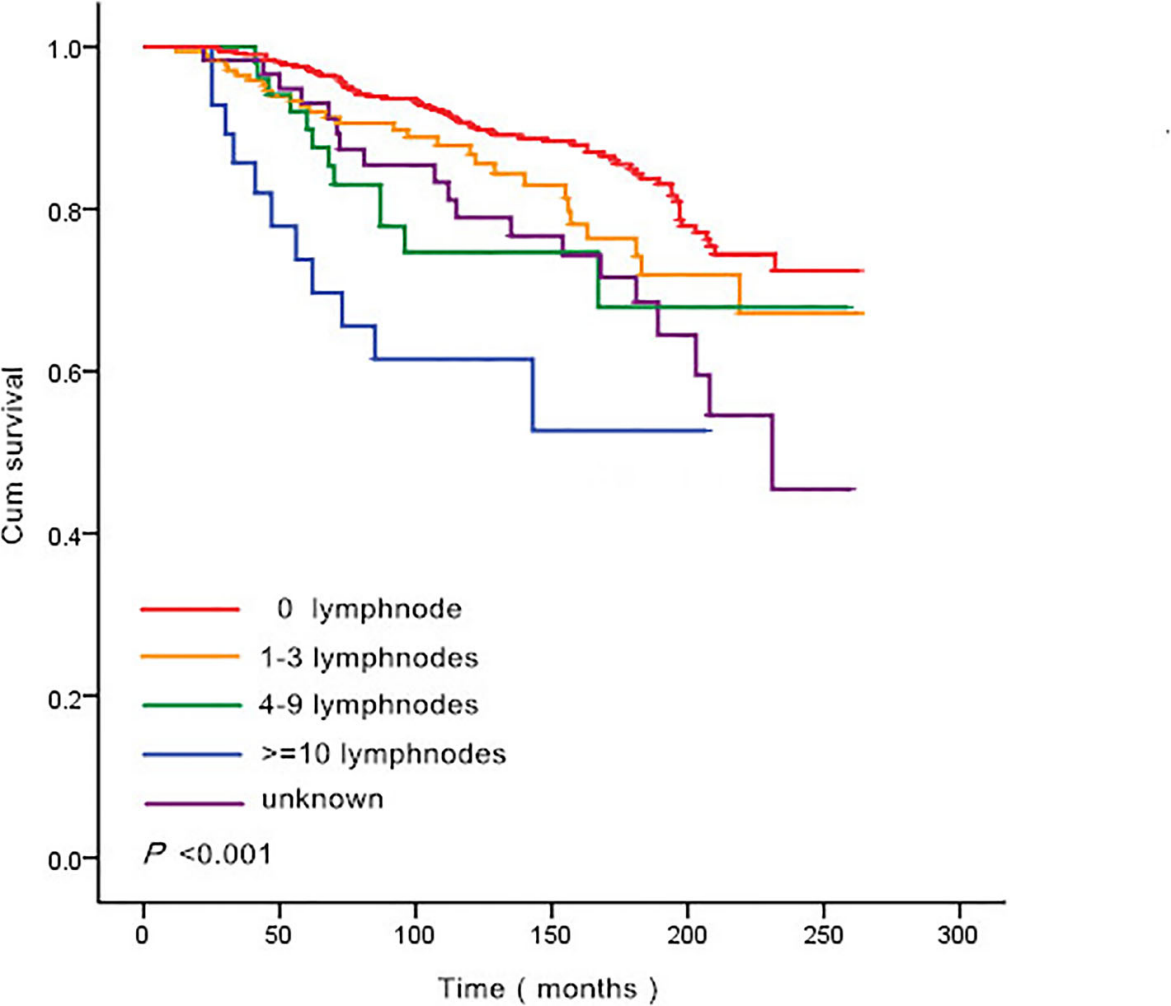
Survival curves for different positive lymph node numbers of 842 breast cancer patients followed by thyroid cancer

In order to determine the lowest cut-off age that could indicate a significantly different prognosis, the contiguous age with different survival was analyzed in SPSS, and 43 years of age was identified as the lowest cut-off age with significantly different prognosis on both sides (*p* = 0.022). Considering the utility in clinic, 50 years was selected as the cut-off age finally, survival curves were summarized in Fig. 1. Two separate OS curves were represented in Fig. 1 when grouped by age 50, patients younger than 50 exhibited a significant advantage for OS. Figure 2 demonstrates the survival curves of different positive lymph node number groups, and 5 separate curves were noted in the figure (*p* < 0.001). With the increase of positive lymph nodes number, a worse prognosis was obtained.

### SIR and SIR influent factors of BC patients followed by TC

**Fig. 3:**
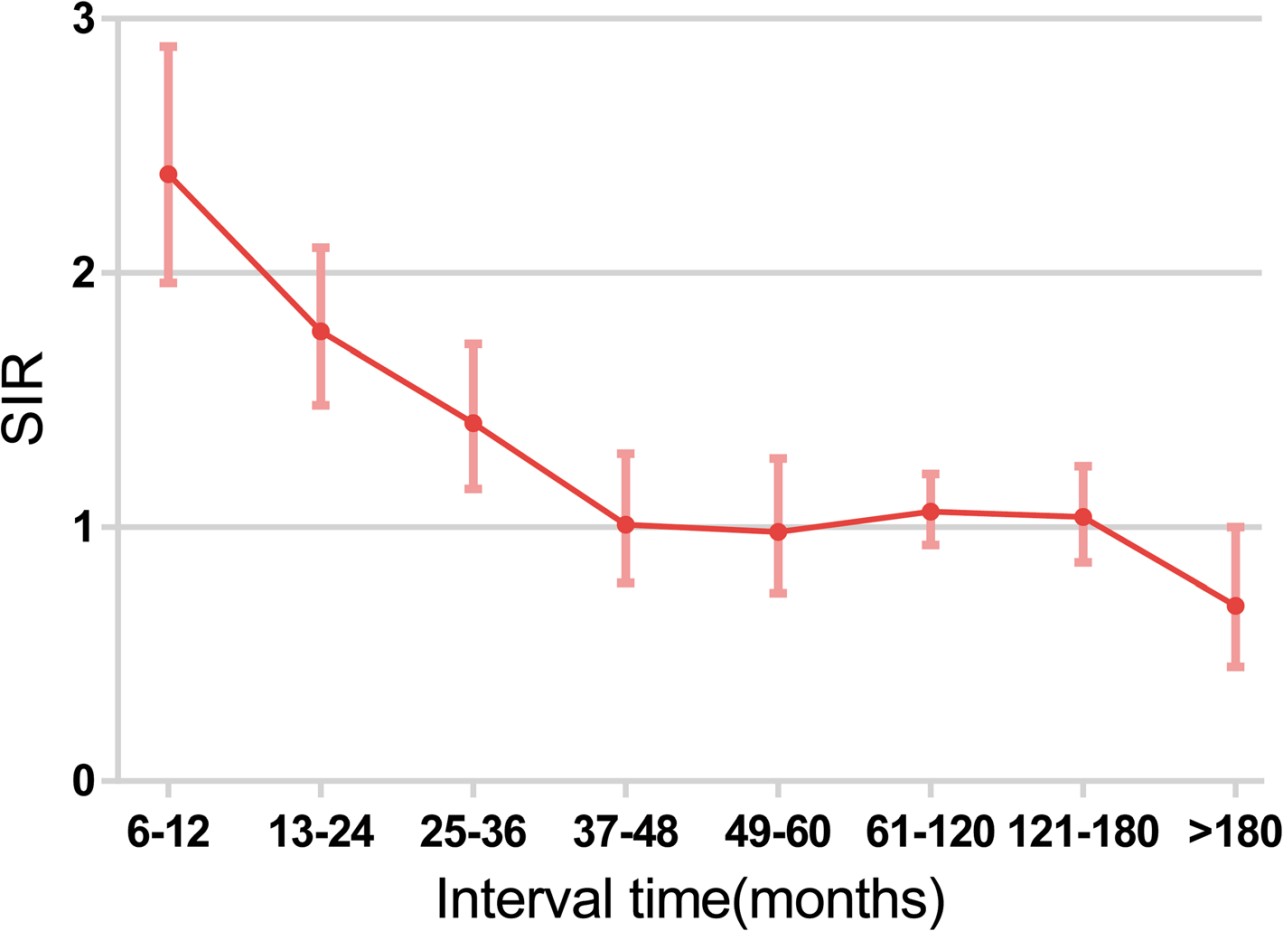
Incidence rate ratios of 842 breast cancer patients followed by thyroid cancer, stratified by time interval

In our study, the SIR was used to compare the incidence rate for the cohort of patients previously diagnosed with BC and subsequently diagnosed with TC to that expected in the general population. SIR> 1 means BC patients have more risk to develop TC than general women. We compared the standardized incidence rate of BC patients with a certain feature with that of general women with the same feature, when the ratio (SIR) > 1 and *p* value<0.05, this feature was defined as a predictive factor, which indicates that BC patients with this feature had an increased probability to further develop TC. The SIR values were generated from the multiple primary-SIR program in SEER*Stat and results are summarized in Fig. 3 and Table 3. The SIR for develop TC was 1.22 (95% CI [1.14, 1.31]) in total.

**Table 3:** Incidence rate ratios of 842 breast cancer patients followed by thyroid cancer, stratified by factors

Variable	Total					6–36 months				> 36 months			
	Observed	SIR	CI low	CI high	Excess risk	Observed	SIR	CI low	CI high	Observed	SIR	CI low	CI high
Race													
White	660	1.15^*^	1.06	1.24	0.37	263	1.67^**^	1.47	1.88	397	0.95	0.86	1.05
Black	77	1.88^**^	1.48	2.34	1.61	32	2.63^**^	1.80	3.71	45	1.56^*^	1.14	2.09
Asian or Pacific Islander	97	1.46^*^	1.18	1.78	1.19	43	2.19^*^	1.59	2.95	54	1.15	0.87	1.51
Site													
Central portion	55	1.48^*^	1.12	1.93	1.13	28	2.76^**^	1.84	3.99	27	1.00	0.66	1.45
Upper-inner	90	1.23	0.99	1.51	0.56	32	1.53^*^	1.05	2.16	58	1.11	0.84	1.43
Lower-inner	44	1.20	0.87	1.62	0.49	17	1.64	0.96	2.63	27	1.03	0.68	1.50
Upper-outer	301	1.19^*^	1.06	1.33	0.46	112	1.65^*^	1.36	1.98	189	1.02	0.88	1.18
Lower-outer	56	1.20	0.91	1.56	0.50	20	1.50	0.92	2.32	36	1.08	0.76	1.50
Overlapping lesion	167	1.19^*^	1.02	1.39	0.48	82	2.04^**^	1.63	2.54	85	0.85	0.68	1.05
Grade													
1	136	1.12	0.94	1.33	0.31	45	1.26	0.92	1.68	91	1.07	0.86	1.31
2	350	1.27^*^	1.14	1.41	0.66	134	1.72^*^	1.44	2.04	216	1.09	0.95	1.25
3	283	1.30^*^	1.16	1.47	0.76	135	2.16^**^	1.81	2.56	148	0.96	0.81	1.12
ER													
Positive	601	1.24^*^	1.14	1.34	0.59	242	1.71^*^	1.50	1.94	359	1.04	0.94	1.16
Negative	155	1.25^*^	1.06	1.47	0.63	72	2.10^**^	1.65	2.65	83	0.93	0.74	1.15
PR													
Positive	532	1.27^*^	1.17	1.39	0.68	216	1.79^**^	1.56	2.04	316	1.06	0.95	1.19
Negative	202	1.15	0.99	1.32	0.36	92	1.78^**^	1.44	2.19	110	0.88	0.71	1.06
ER (+) PR (+)	507	1.26^*^	1.15	1.37	0.64	206	1.75^**^	1.52	2.01	301	1.05	0.94	1.18
ER (−) PR (−)	125	1.16	0.96	1.38	0.40	61	1.97^*^	1.51	2.53	64	0.83	0.64	1.06

*SIR* Standardized incidence ratio, *CI* Confidence interval, *ER* Estrogen receptor, *PR* Progesterone receptor

*p < 0.05; **p < 0.01

The mean latency from diagnosis with BC to the development of TC was 65.7 months. The incidence of developing primary TC was significantly high within the first 3 years of BC diagnosis and then decreased, with no significantly difference from that of the general population (Fig. 3). Therefore, a latency of 3 years was used as the cut-off when evaluating the influence of other factors in Table 3.

Different influent factors were summarized in Table 3, when SIR > 1 and *p* value<0.05, this factor was defined as a predictive factor, which indicates that BC patients with this feature had an increased probability to further develop TC. When considering race, white, black, Asian, and Pacific Islander patients all had significant high SIRs. Black women with BC had the highest SIR of 1.88, and even beyond the 3-year latency, the risk was still higher in these patients to develop TC than in general black women (SIR 1.56, 95% CI [1.14, 2.09]). The excess risk of black women was 1.61 per 10,000 persons, which was the highest in all SIR influent factors. With regard to the site of BC, tumors located in upper-outer quarter or central portion or overlapping lesions showed a significant increase in the risk of TC development (SIRs were 1.48, 1.19, and 1.19, respectively). BC patients with high grade (grade 2 or 3) tumor also had significantly higher risk to develop TC. With regard to the status of hormone receptors, BC survivors were at a significantly high risk for developing TC when both the estrogen receptor (ER) and progesterone receptor (PR) were positive (SIR 1.26, 95% CI [1.15, 1.37]). But when only considered the SIR within 3 years of BC diagnosis, all BC survivors had a significantly high incidence to develop TC irrespective of ER or PR status.

## Discussion

The possibility of a relation between BC and thyroid diseases has been considered for several years, and the foundation of this opinion is the breasts and thyroid are hormone responsive organs, which are closely related to endocrine function changes and glandular diseases [11]. One of the first studies to suggest this relationship was the study by Beatson [12] in 1896, in which thyroid extract was used in the treatment of BC. Then, studies have evaluated the risk of developing second primary malignancies in patients with BC or TC history [2, 4–6], mainly concentrate on the influence of radiotherapy. Some studies demonstrated that BC patients have more chance to develop second primary TC and vise versa [3, 7, 9]. Few studies have focused on the features and survival of BC survivors with TC and the factors that influence the risk of BC survivors to develop TC. Our study is novel in that it focused on the clinicopathological features, survival, and predictive factors of BC survivors who developed primary TC, based on the cohorts from the SEER database.

Table 1 shows that patients with both BC and TC were more likely to be white, have a median age of 54 years, have a tumor size less than 2 cm, have no lymph nodes metastasis, show infiltrating duct pathology, show grade 2 or 3 histology, and with hormone receptors positivity. In a previous study, An et al. [3] investigated 81 BC patients followed by TC, median age at diagnosis of BC was 43.4, 86.5% BC were infiltrating duct carcinoma and ER, PR expressions increased significantly in BC patients. The present results are consistent with these above. Additionally, compared the clinicopathological features and prognosis with those of BC only patients, BC patients followed by TC were younger, had a significantly smaller tumor size, and had a relatively better prognosis. An earlier age at diagnosis, a smaller tumor and a well prognosis increased the chance of developing a second tumor in the following years, which few studies have mentioned before. With regard to the better prognosis of BC survivors with TC, it mainly due to the early age at diagnosis, the 10-year survival of TC patients reaching 95–97% [9] was another reason. Considering the factors that influence the survival of patients with both BC and TC, age at diagnosis of BC and the number of positive lymph nodes were identified after adjustment, and these are known to be prognostic factors in BC only patients [13], seeming there is no much difference existed. As a clinician, by using these prognosis factors, we are able to identify patients who may get a worse survival, which give us a chance to make effort to improve their prognosis at the time of diagnosis or during patients’ therapy.

The SIR for develop TC was 1.22 (95% CI [1.14, 1.31]) in total, results from other studies range from 1.28 to 2.18 [3, 7, 14, 15]. The significantly high SIR indicated BC patients had an increased risk for developing TC, which was not just the result of increased medical surveillance for breast cancer. Hormonal, genetic, and environmental factors, and treatment modalities might play roles in this co-occurrence [3, 9, 14, 16]. Moreover, a common molecular mechanism might exist, rather than an incidental association. Researchers proposed ER/PR signaling might represent common etiological factors in the development of TC and BC, studies on the mechanism, including the ER pathway in thyroid tissues and mutations in the CHEK2 gene, have been reported previously [3, 14, 17]. Clinicopathological features in BC then TC group also showed a tendency in more positive expression of ER/PR than BC only group in Table 1. Several articles evaluating radiotherapy after BC diagnosis as a possible cause of the increased risk of second primary TC. Grantzau [4] conducted a meta-analysis of 762,468 BC patients and found radiotherapy for breast cancer is associated with a small but significantly increased risk of second cancers of the lung, esophagus, and soft tissues, but no significant association between radiotherapy and second thyroid cancer. Another research [14] containing 55,318 women from the National Health Insurance system of Taiwan, compared the risk of TC among BC patients who received radiotherapy or not, the risk to develop TC in women who received radiotherapy was not significantly higher than those not. Some other research based on different nations agreed with the opinion that radioactive iodine therapy did not significantly increase the incidence of subsequent BC [18–20]. But a research by Wei Lin [21] showed the risk of TC did increase in some BC subgroups, adjusted hazard ratios of TC risk for women receiving radiotherapy was 1.97 among BC survivors aged older than 55 years, 1.93 among BC survivors without chemotherapy. Though radiotherapy may increase the risk of developing second TC in some subgroups of BC survivors, its role in BC treatment and the beneficial on OS overweight the risk for developing second TC.

The mean latency from diagnosis with BC to the development of TC was 65.7 months, this interval time was 5.6 years in the research of Garner et al. [9] and 5.9 years (range 3.4–12.7 years) in the research of An et al. [3]. A time interval of 3 years is essential for BC patients, for the risk of TC development was significantly higher within 3 years, with SIR of 2.39 (95% CI [1.96, 2.89]) in the first year. An et al. [3] observed the incidence of second primary TC was significantly higher within the first 5 years of BC diagnosis, and then decreased over time, in 81 BC then TC patients. The trend of SIR was same with our results, the difference in the cut-off year may be the result of different sample size. This result prompted consideration about the screening and treatment of individuals with the co-occurrence of these two diseases.

Screening is an approach to detect a disease early in asymptomatic individuals, but it may lead to a more aggressive operation. As a result, it is important for clinicians to recognize high-risk individuals with a BC history. Our study indicated to some degree that black women with BC should be focused on, even 3 years after BC diagnosis. Besides, a primary tumor located in an upper-outer, central, or overlapping site should also be carefully assessed, high tumor grade and histology with both ER and PR positive expression are other important features. BC patients with these features have exceeded risk to develop TC. As a clinician, when making medical decision for BC patients with these predictive factors, we should avoid to choose therapeutic methods those may increase the incidence of TC, which was the meaning for us to recognize these factors. In the previous study, Marcheselli et al. [2] demonstrated that human epidermal growth factor receptor 2 (HER-2) positivity, and BRCA1 or BRCA2 mutation had an effect on the risk of second primary neoplasms, but the effect was not limited to TC. These SIR influent factors in our manuscript are new with regard to the relationship between BC and TC. Regarding to the intensity of screening, as the highest excess risk was 1.61/10,000 in black women, a routine screening intensity is sufficient, however, clinicians should still consider the possibility of further developing TC in BC patients, especially individuals with black race, upper-outer or central site tumor, high tumor grades, and both ER PR positive expression.

The present study demonstrated the clinicopathological features and survival of 842 BC patients followed by TC, represented a higher risk of TC development in BC patients than in the general population, especially within 3 years, and the predictive factors of BC patients developing TC. The present study had some limitations. First, although population-based cancer registries provided a lot of information to the SEER database, data on HER-2 status were recorded only since 2010; therefore, among the 842 patients, only 68 had HER-2 records, which prevented the identification of the molecular subgroup of BC and may cause bias on the analyzing of HER-2. Second, this study was based on data from a database rather than data from research in a clinic, which might reduce the level of evidence. Further studies in the clinic and laboratory should be conducted to reveal more insights on the relationship of BC and TC.

## Conclusions

BC patients who subsequently developed TC were more likely to be white and at a middle age, diagnosed with more T1 tumor, less lymph nodes metastasis, more infiltrating duct pathology, more 2 or 3 grade, and more hormone receptors positive disease. Compared with the features of BC only patients, they were younger, had a smaller tumor size, and had a relatively better prognosis. Additionally, age at the diagnosis of BC and the number of positive lymph nodes were prognostic factors to the OS of BC patients followed by TC after adjustment. Moreover, BC patients had a high risk of developing TC (SIR 1.22), especially within 3 years. Black women, primary tumor located in an upper-outer, central, or overlapping site, high grade tumor and with positive hormone receptors expression were risk factors of SIR, BC patients with these features showed an increased risk of TC development.

## Data Availability

The datasets analyzed during the current study are available in the SEER*Stat software (version 8.3.6, download from https://seer.cancer.gov/data/options.html). A registration form needs to be completed before using and filter criteria need to be added. The datasets are also available from the corresponding author on reasonable request.

## Abbreviations

BC: Breast cancer
CI: Confidence interval
ER: Estrogen receptor
HER-2: Human epidermal growth factor receptor 2
OR: Odds ratio
OS: Overall survival
PR: Progesterone receptor
SEER: Surveillance, Epidemiology, and End Results
SIR: Standardized incidence ratio
TC: Thyroid cancer
